# Radiocarbon dating minute amounts of bone (3–60 mg) with ECHoMICADAS

**DOI:** 10.1038/s41598-017-07645-3

**Published:** 2017-08-02

**Authors:** S. Cersoy, A. Zazzo, J. Rofes, A. Tresset, S. Zirah, C. Gauthier, E. Kaltnecker, F. Thil, N. Tisnerat-Laborde

**Affiliations:** 1 Archéozoologie, Archéobotanique: Sociétés, Pratiques et Environnements (AASPE, UMR 7209), Sorbonne Universités, Muséum national d’Histoire naturelle, CNRS, CP55 ou 56, 55 rue Buffon, F-75005 Paris, France; 2 Laboratoire des Sciences du Climat et de l’Environnement LSCE/IPSL, CEA-CNRS-UVSQ, Université Paris-Saclay, F-91191 Gif-sur-Yvette, France; 3 Molécules de Communication et Adaptation des Microorganismes (MCAM, UMR 7245), Sorbonne Universités, Muséum national d’Histoire naturelle, CNRS, CP 54, 57 rue Cuvier, F-75005 Paris, France

## Abstract

Because hard tissues can be radiocarbon dated, they are key to establishing the archaeological chronologies, palaeoenvironmental reconstructions and historical-biogeographical processes of the last 50,000 years. The advent of accelerator mass spectrometers (AMS) has revolutionized the field of archaeology but routine AMS dating still requires 60–200 mg of bone, which far exceeds that of small vertebrates or remains which hold a patrimonial value (e.g. hominid remains or worked bone artefacts). Here, we present the first radiocarbon dates obtained from minute amounts of bone (3–60 mg) using a MIni CArbon DAting System (MICADAS). An optimized protocol allowed us to extract enough material to produce between 0.2 and 1.0 mg of carbon for graphite targets. Our approach was tested on known-age samples dating back to 40,000 BP, and served as proof of concept. The method was then applied to two archaeological sites where reliable dates were obtained from the single bones of small mammals. These results open the way for the routine dating of small or key bone samples.

## Introduction

Hard tissues (i.e. bones, teeth, antler and ivory) found in the fossil record have a tremendous informative potential relevant to the fields of archaeology, palaeoecology and the history of art and technology. Because they can be identified to the species level and radiocarbon dated, these fossil remains are key to establishing the archaeological chronologies, palaeoenvironmental reconstructions and historical-biogeographical processes (i.e. post-glacial recolonization events) of the last 50,000 years. In effect, they provide us with windows to past societies, and contribute to our knowledge of ancient human evolution and cultural development^1^, palaeoclimates^2^, paleoenvironments^3^ and past trade networks^4^. Hard tissues contain an organic phase (mainly the protein collagen type I) embedded in a mineral phase (made of a non-stoichiometric biogenic apatite). While the exchange of inorganic carbon occurs much more readily^5, 6^, the relative chemical inertness of biopolymers makes them ideal for dating; therefore, the majority of bone radiocarbon dates are obtained from the collagen phase. The chemical integrity of this biomolecule can be assessed using simple biochemical criteria such as %C, %N and C/N ratio^7–9^. The amount of collagen in fresh bone is approximately 20–25%^9, 10^. As the diagenetic alteration proceeds, the quantity and quality of the collagen decreases; consequently, the sample size must increase in order to compensate for protein loss. Radiocarbon dating ancient bones can therefore prove challenging. The advent of accelerator mass spectrometers (AMS) in the eighties revolutionized the field of archaeology by allowing smaller samples to be measured. While it decreases the amount of carbon required for a radiocarbon measurement by several orders of magnitude, the AMS dating of bone collagen still requires at least 60–200 mg of bone^11–13^, depending on the protein preservation and the extraction protocol. However, this is still excessive for two classes of bone remains: (1) individual bones of small vertebrates which often weigh less than 60 mg; and (2) unique remains such as hominid bones or worked bone artefacts for which curators do not permit invasive sampling^14^.

The specification of sample weights used for dating is not considered necessary by the scientific community^15^ and is seldom reported in publications, even when supplementary information is available (see for example refs 16–19). However, careful examination of the literature suggests that attempts at dating samples smaller than 60 mg are rare. Regarding small vertebrates, only two case studies were found: the Late Prehistoric dispersal of Polynesians to New Zealand was dated using the commensal Pacific rat as a proxy^20^, and the time scale of the collared lemming’s (*Dicrostonyx* spp.) recolonization of Eurasia and North America was established through direct bone dating^21^. In both studies, the bones were Late Pleistocene to Holocene in age, and weights were comprised of between 30–60 mg. Regarding hominid fossils (i.e. bone and teeth) and their associated tools (i.e. ivory, bone or antler), progress in sample pretreatments using ultrafiltration^22^, have led to the redating of samples initially dated during the eighties and nineties^23–27^. However, ultrafiltration is often associated with lower extraction yields (especially when bones are moderately to poorly preserved), and does not always allow for the recovery of a sufficient amount of collagen when sample mass is lower than 100 mg. As a result, all previous attempts have failed^23, 24, 28^. In general, the solution consists in dating a “reliably associated” artefact (often charcoal) from the same stratigraphic unit instead of the bone remains.

The main consensus in the radiocarbon community is that bones with less than a 1% collagen yield should not be dated^9, 29^. Consequently, what defines the initial amount of bone material needed for a reliable ^14^C measurement is the 1% collagen yield threshold. Considering that collagen contains about 40–45% carbon, 250 mg of bone are necessary to provide enough carbon for a regular-sized graphite target of 1 mg. For well-preserved bone (20–25% collagen), the sample size decreases to about 10 mg.

In practice, the manipulation of small bone samples presents several obstacles which are difficult to overcome, especially for ancient (Middle to Upper Paleolithic transition) samples. Recent advances in graphite sample preparation and AMS capabilities make it possible to now run very small samples (<0.1 mgC) using graphite targets^12, 30, 31^. These methods are complex and labour intensive as they require adaptation of the graphitization procedures and the running of multiple standards and samples of identical size to account for increased risks of contamination. Moreover, the effect of the blank correction on ^14^C results increases exponentially as samples decrease in size. For these reasons, AMS dating using a very small graphite (<0.1 mgC) target has not become routine for archaeological samples. An alternative solution is to cut the graphitization step by using a gas ion source of the small AMS^32^. This idea is not new, but recent advances in technology suggest that the stability and reproducibility achieved by the new compact AMS, like the MIni CArbon DAting System (MICADAS), could make them suitable to run very small (<0.1 mgC) samples with acceptable accuracy. This method has been tested on gaseous samples obtained from carbonate, particulate organic carbon and aerosols^33–35^ but not on compound specific material such as bone collagen. However, with decreasing sample sizes comes an increased risk of contamination from the burial environment and from laboratory handling. Moreover, due to lower counting statistics, precision is usually much lower with the gas ion source than with the graphite target (2% vs. 0.3%) resulting in uncertainties that are unacceptable for most archaeological samples. They can be run in triplicates in order to improve the precision, but this requires the initial sample size to be increased, thus decreasing the interest of the gas ion source for archaeological samples.

Here, we present the first radiocarbon dates obtained from minute amounts of bone (3–60 mg) using ECHoMICADAS, the compact AMS^36, 37^ recently installed at Gif-sur-Yvette, France. The optimization of our bone collagen extraction protocol allowed us to decrease the sample size by two orders of magnitude, while still extracting enough material (>0.2 mgC) to use the automated AGE 3 graphitization device^38^. Our approach was elaborated on known-age samples from the Fifth International radiocarbon Inter-comparison (VIRI) and served as proof of concept. The method was then applied to two archaeological sites where the single bones of small mammals were AMS-dated, and the dates compared to standard-size bone samples found in the near vicinity.

## Results and Discussion

### Known-age samples

Collagen was extracted from four macromammal bone samples of known-age (for details please refer to the Methods section) covering the full range of radiocarbon dating: a horse bone VIRI F (less than one ^14^C half-life), two whale bones VIRI I and VIRI H (approximately two half-lives) and a mammoth bone VIRI E (more than five half-lives). The efficiency of eight collagen extraction protocols (in terms of quantity of protein extracted and collagen integrity) was tested on seven samples in a previous study^39^. Four representative protocols were tested here and are summarized in Table 1: a soft protocol (B)^40, 41^ which appeared to be the most appropriate to recover enough collagen from micromammal bone samples^39^, two intermediate protocols (C^42^ and E^43, 44^) involving ultrafiltration of the collagen extracts and a harsher protocol (F)^45^ currently used in our laboratory for the radiocarbon dating and isotopic analysis of macrovertebrate bone samples. The results obtained for the four known-age (VIRI) bone samples are summarized in Supplementary Table S1. The impact of sample size on the collagen extraction yield and the radiocarbon age are discussed below.

**Table 1:** Collagen extraction protocols: summary of the different steps. Protocols were designated in agreement with ref. 39.

**Protocol**	Crushing	Demineralization		Decontamination	Gelatinization step			Purification steps	
	Size	Agent	Duration	Duration	pH	Temperature	Duration	Filtration (pore size)	Ultrafiltration (cut-off)
B	5–10 mm	HCl 0.2 M (4 °C)	2–4 days	Yes (4 °C)	1	90 °C	5 min–1 h	Glass (1.6 μm)	No
C	Chunks (10–60 mg)	HCl 0.25 M	Several days	No	2	58 °C	16 h	Ezee^TM^ (45–90 μm)	Yes (30 kDa)
E	Coarsely ground chunks	HCl 0.5 M	24 h	30 min	3	75 °C	20 h	Ezee^TM^ (45–90 μm)	Yes (30 kDa)
F	Powder 0.3–0.7 mm	HCl 1 M	20 min	20 h	2	100 °C	17 h	MF-Millipore (5 μm)	No

#### Impact of sample size on collagen yield

**Figure 1:**
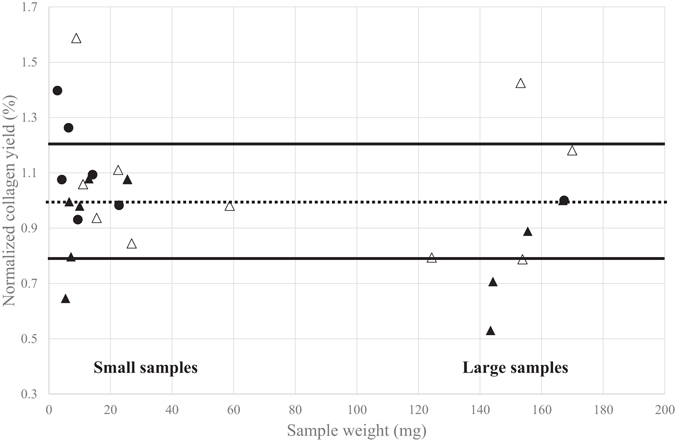
Relationship between sample size and collagen yield. Open circles: VIRI F horse bone sample, closed circles: VIRI I whale bone samples, open triangles: VIRI H bone samples and closed circles: VIRI E bone samples. The yield was normalized based on the yield obtained from large sample amounts (>100 mg) using protocol F. The dotted line indicates the normalized yield. The solid lines indicate the one sigma standard deviation for large samples prepared using different extraction protocols.

The impact of sample size on collagen extraction yield is shown in Fig. 1. High yields (between 15 and 20%) were obtained, in accordance with results previously reported for these bones (e.g. in ref. 46). In this figure, a normalized yield was calculated for clarity and to enable direct comparisons. It corresponds to the ratio of the final estimated yield over the median value of the collagen yield obtained for large samples (>100 mg) using the in-house F protocol^45^ (23.9% for VIRI F, 22.8% for VIRI I, 16% for VIRI H and 19.8% for VIRI E). Overall, normalized extraction yields for small (<100 mg) samples are similar to those for large (>100 mg) samples. We observed an inter-individual variability, but it was of the same order of magnitude for small and large samples (one sigma standard deviation 0.2). Moreover, yield variations did not correspond to abnormal %C or %N or to C/N ratios (see Supplementary Table S1). This could be due to errors in weight estimates (especially for small samples) and/or local heterogeneities of collagen preservation in these particular bones. These results suggest that the protocol designed for very small samples is efficient at recovering enough collagen for radiocarbon dating.

#### Impact of the preparations on radiocarbon age

Following collagen extraction, all samples were wrapped in tin capsules. For practical reasons (imperceptibility and electrostaticity) the collagen extracted from small samples could not be transferred in its solid state; therefore, it was resuspended in ultrapure water prior to being introduced into the tin capsules and evaporated on a hot plate. To check whether this extra step had an effect on the radiocarbon age, age distributions for both types of preparations (solid and liquid) were compared using the non-parametric test of Wilcoxon-Mann-Whitney^47^ with a one-sided risk of 1%. The two distributions were identical for all the VIRI samples (U = 67, 22, 109, 106 for VIRI F, VIRI I, VIRIH and VIRI E, respectively) demonstrating that the age quality of the results was the same for both preparations.

#### Impact of carbon mass on radiocarbon age

**Figure 2:**
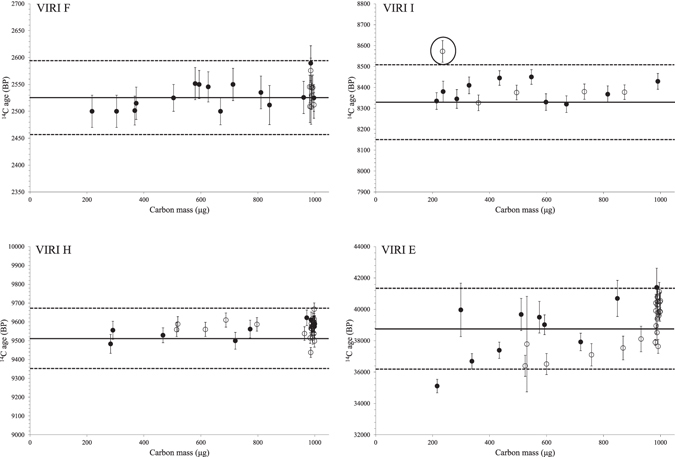
Relationship between the carbon mass of the graphite target and the measured radiocarbon age of the VIRI samples. Solid lines correspond to the average consensus AMS age for each VIRI sample. Dotted lines indicates the upper and lower limit of consensus AMS ages (one sigma, as stated in ref. 48). Open and closed circles are for large (>100 mg) and small (<100 mg) bone samples, respectively. The one encircled VIRI I sample is an outlier as checked by the Dixon test. The age variability observed for VIRI collagen, prepared using different extraction protocols, is comparable with the one reported in ref. 46.

Depending on sample size and extraction yields, graphite targets with a carbon mass of between 0.2 and 1.0 mgC were obtained. Radiocarbon ages were plotted against the carbon mass for each sample (Fig. 2). We tested the effect of carbon mass on the radiocarbon age using the Spearman’s rank correlation coefficient^47^ with a one-sided risk of 1%. No correlation was observed for the Holocene samples (ρ = 0.2503, −0.0607, and 0.3623 for VIRI F, VIRI I and VIRI H, respectively). All the ^14^C results from these VIRI were in agreement with the consensus values (see Supplementary Table S1). A significant correlation was observed for the Pleistocene mammoth sample VIRI E (ρ = 0.5884). Decreasing radiocarbon ages were measured for the small samples, suggesting an increasing contribution of contamination with modern carbon. This tendency was observed for the small samples introduced in liquid form (ρ = 0.6242) into the tin capsules, but also for samples introduced as solids which had been prepared from large amounts of bone (ρ = 0.9030 for F protocol). Therefore, the correlation between age and carbon mass seems to indicate an insufficient blank correction for the smallest masses (0.2–0.5 mgC), rather than contamination during the collagen extraction procedure. We are planning to use bone blanks in the future (using SIRI bone C, for example), in order to better determine the magnitude of the background correction factor that should be applied to ^14^C results for small samples near the limit of radiocarbon dating. Despite this trend, which was only observed for the Pleistocene mammoth sample, the measured ages always fell within the consensus age limits performed in other AMS labs^48^ (see Methods section and Supplementary Table S1) and are not statistically different (P’ 0.05, Student’s test).

Overall, our results show that reliable ages can be measured on bone samples with a carbon mass as low as 0.2 mg C. Considering the 1% collagen yield threshold, and the concentration of carbon in collagen, this result indicates that it is possible to routinely date bone samples weighing between 3 and 60 mg, depending on the amount of collagen present. It is noteworthy that the bone collagen yield can be estimated prior to collagen extraction using FTIR spectroscopy^39, 49^. This technique is fast and requires only 1 mg of bone powder. Prescreening using FTIR spectroscopy allows for the adjustment of the sample size, thus minimizing the damage to the sample and avoiding sampling if collagen preservation is too low.

### Application to archaeological samples

The collagen extraction method designed for small samples was applied to five archaeological micromammal individual bone samples weighing between 15.6 and 22.8 mg. Results are reported in Table 2. Despite a relatively poor quality of collagen preservation (yields ranging from 2.8 to 9.9% as opposed to 13.1 to 21.3% for the VIRI), and the small size of the samples, we were able to extract enough collagen (0.231 to 0.700 mgC) to date all the samples. C/N ratios ranged between 3.2 and 3.5, well within the 2.9–3.6 limits, indicating that the extracted collagen was suitable for radiocarbon dating.

**Table 2:** Radiocarbon dating of archaeological samples.

Site	Species	Element	Sample prep code	ECHo n°	Sample size (mg)	%C	%N	C/N	Carbon mass (μgC)	Amount collagen (mg)	Yield (%)	^14^C age (BP)	error
Peyrazet	*Neomys anomalus*	Left hemimandible	16291	1254.1.1	22.8	30.7	10.2	3.5	266	0.64	2.8	12960	70
	*Neomys anomalus*	Right hemimandible	16290	1253.1.1	15.6	24.3	8.4	3.4	231	0.55	3.6	12940	70
Bourges	Small carnivore	Long bone	16269	1237.1.1	17.0	35.9	12.9	3.2	700	1.68	9.9	2270	25
	Rodent	Tibia	16271	1243.1.1	17.2	27.9	9.9	3.3	358	0.86	5.0	2310	30
	Shrew (*Crocidura*)	Right hemimandible	16295	1257.1.1	22.6	33.9	12.0	3.3	504	1.21	5.4	2310	30

Samples characteristics, origin and zooarcheological identifications are addressed. Sample size, graphitization results and radiocarbon dates are also reported for each sample. Yield is estimated as the ratio (in percent) of the total amount of collagen recovered from the amount of initial bone used for extraction. The carbon mass corresponds to the amount of carbon detected following combustion in the Elemental Analyser and used to produce the graphite target. ECHo n° corresponds to the target numbers.

**Figure 3:**
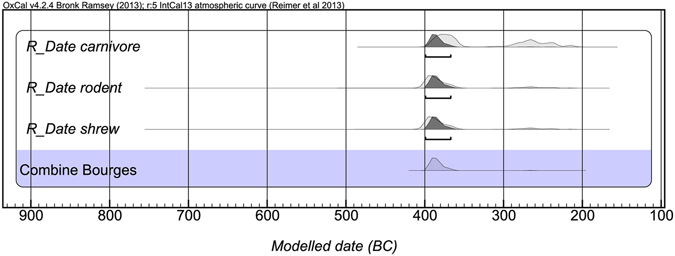
Calibration and Bayesian modeling of the three AMS dates on small mammal samples from Bourges (France). Dates were modelled in OxCal v.4.2.4^56^ using IntCal13 atmospheric curve^57^.

In the case of the first archaeological site (Bourges), the three dates obtained from the three different species (i.e. a rodent, a small carnivore and a bicolored white-toothed shrew) were compatible. Calibrated plots are shown in Fig. 3. The calibrated age (400–360 cal BC at 2σ with T’ = 1.488 < 5.990, chi-square test), was also in agreement with the estimated relative age of the stillage storage deposit (Late Iron Age, La Tène) as described in ref. 50.

In the second study, the dates obtained from the Late Pleistocene Miller’s water shrews from the Magdalenian layers of the Peyrazet site yielded statistically identical results (12960 ± 70 and 12940 ± 70 BP, respectively). These results can be compared with an AMS date obtained using a mix of rodent bones (genus *Arvicola*) from the same stratigraphic unit^51^. This date (12960 ± 70 BP, Lyon-11974/SacA40416), fits perfectly with those obtained on the two single shrew hemi-mandibles (T’ = 0.054 < 5.990), using a sample at least ten times smaller.

These two case studies illustrate the robustness of this method for the accurate dating of small amounts of archaeological bone samples dating back to the Late Pleistocene.

This work demonstrates that it is possible to obtain reliable radiocarbon dates from very small amounts of bone (less than 10 mg) ranging in age from 0 to 40,000 BP. All the samples provided enough collagen (>0.2 mg C) to allow dating on graphite targets. No effect on the measured age due to sample size was documented on the Late Pleistocene or Holocene samples. For the oldest (VIRI E) mammoth sample, a possible effect was noted for carbon masses smaller than 0.5 mg C, possibly due to an insufficient blank correction. Further work is planned to improve blank correction for small samples near the limit of radiocarbon dating. Our results show that reliable radiocarbon dates can be measured on mid- to well-preserved bones samples, using as little as 3 mg of bone. These results open the way for the routine radiocarbon dating of small bone samples. Research projects using small mammals as palaeoenvironmental and/or historical-biogeographical proxies or as proxies for human-animal interactions (i.e. dispersion of commensal micromammals) are already ongoing in our laboratory, and direct dating performed on micromammal remains will be crucial for establishing precise and correct chronological frameworks. The radiocarbon dating of unique bone, ivory or antler artefacts (e.g. perforated batons, Venuses from the European Upper Paleolithic or emblematic fossil human remains) can now also be seriously considered.

## Methods

### Sample selection

Four reference macromammal bone samples from the Fifth Radiocarbon Inter-comparison (VIRI) procedures which spanned the full range of radiocarbon dates, were used to test the methodology: a horse bone from a Scythian burial in Siberia (VIRI F, 2525 ± 69 BP, n = 38), two whale bones from Svalbard, Norway (VIRI I, 8328 ± 176 BP n = 41 and VIRI H, 9510 ± 158 BP n = 38) and a Pleistocene mammoth bone from the Yukon Territory, Canada (VIRI E, 38772 ± 2532 BP n = 40). Consensus values for the ages were determined from about forty measurements involving forty-two ^14^C laboratories (for details, see ref. 48).

Five archaeological samples from two different sites, weighing between 15.6 and 22.8 mg, were also selected. Two Miller’s water shrew (*Neomys anomalus*) mandibles were chosen from among the microfaunal assemblage from the Upper Magdalenian levels of the cave-shelter site of Peyrazet (Dordogne, France)^52^. Three bone samples were also selected from among the microfaunal assemblage of a collective inhumation deposit dating to the Iron Age (La Tène B2/C1 period), located in Bourges (Port Sec south site), France^50^. They included a rodent tibia, a small carnivore long bone and a right mandible from a bicolored white-toothed shrew (*Crocidura leucodon*). These samples are part of a broader research project, carried out in our laboratory, aiming, inter alia, at reconstructing the post-glacial recolonization process of several species of shrews in western Europe (Marie Curie Fellowship MCA-IEF FP7/2007–2013 Project no. 629604).

### Collagen extraction

VIRI bone samples were crushed into fragments and the collagen extracted using the various protocols described in ref. 39 and summarized in Table 1: a protocol optimized for microsamples after Stafford^40^ and Waters *et al*.^41^, was chosen for small samples (<100 mg) to recover sufficient collagen for radiocarbon dating. Briefly, bone shards (5–10 mm) were demineralized in 0.2 M HCl at 4 °C for 2 to 4 days (visual and mechanical check). The remaining translucent “*phantom*” (acid decalcified collagen) was immersed in 0.1 M NaOH at 4 °C for 2 to 4 days for decontamination. Finally, gelatinization was performed in 0.06 M HCl at 90 °C for up to 1 hour, followed by glass filtering. Chemicals of ACS grade (hydrochloric acid 37% and sodium hydroxide pellets), were purchased from Sigma-Aldrich (France) and all solutions were freshly prepared. To minimize laboratory contamination during extraction processing, borosilicate glassware (flasks, 10 mL centrifuge tubes and glass Pasteur pipettes) underwent special cleaning including boiling in diluted Decon 90 surfactant (Decon Laboratories Limited, France), rinsing with twice-distilled water, overnight immersion in 10% hydrochloric acid (technical grade, Sigma-Aldrich, France), rinsing with bidistilled water and heating to 450 °C for 5 hours to remove any possible organic contamination. Glass pieces were stored in aluminum foil until use to prevent dust from entering. Likewise, laboratory benches were covered in aluminium foil during sample processing and were regularly replaced; nitrile disposable gloves and clean laboratory coats were worn at all times. Bone microsamples varied in size from 58 down to 3 mg. To allow comparison, large amounts (100–500 mg) of VIRI bone samples were also prepared according to the three standard collagen extraction protocols available in the scientific literature^39^: the usual bone preparation procedure performed in our laboratory^45^ and two protocols involving ultrafiltration^42–44^. All the protocols were labelled according to their designation in ref. 39: Stafford (B) for the soft Stafford *et al*.^40, 41^ protocol, Brown (C) for the Brown *et al*.^42^ protocol, ORAU(E) for the Brock *et al*.^43, 44^ protocol and MNHN (F) for the Bocherens *et al*.^45^ protocol. Collagen from the archaeological samples was extracted using the same optimized protocol as for the VIRI microsamples and was applied directly on the intact mandibles. Collagen extracts were freeze-dried for at least 48 hours. For large (100–500 mg) samples, the extraction yield was estimated by weighing the collagen on an analytical balance (sensitivity: 0.1 mg). For the smallest samples, the yield was estimated based on the amount of graphitized carbon, assuming an average carbon concentration of 42% in bone collagen.

### Sample combustion and graphitization

Collagen samples were wrapped in 5 × 9 mm low ^14^C content tin capsules (Säntis Analytical AG, Switzerland). For large samples, the required amount of solid collagen (about 2.5 mg) was directly introduced into the capsule using dedicated handling tools and spatulas, thoroughly sterilized with 95% ethanol and kept in aluminum foil between each preparation sequence. For small VIRI and archaeological samples, collection in a solid state was impossible and another procedure was developed. Freeze-dried extracts were resuspended in fresh Type I (ultrapure) water and encapsulated in a liquid state, then evaporated on a heating plate at 110 °C for 2 hours. Tin capsules were prepared using both procedures, weighted on a microbalance (precision: 5 μg), closed and folded.

They were then combusted in the elemental analyzer (EA) of a commercially available AGE 3 (Ionplus, Switzerland) automated compact graphitization system^38^. The quality control parameters (%C, %N and C/N ratios) reported here were measured in the EA prior to graphitization and did not require an extra sample to be taken. The CO_2_ was then transferred to the graphitization unit where the process was performed in the seven reactors with 5 or 3 mg of iron catalyst for large (0.4–1.0 mg C) and small (0.2–0.4 mg C) samples, respectively, so as to keep the Fe/C ratio above 5^12, 53, 54^. In order to reduce the risk of memory effects in the graphite reactors, a sample of the same expected age was combusted prior to each test or archaeological sample. Graphite samples were then pressed into targets within a few days. Two oxalic acid II standards and two phtalic anhydride blanks were processed every ten samples^36^.

### AMS measurements and data reduction

Graphite targets were dated using the ECHoMICADAS AMS at Gif-sur-Yvette (France). Data reduction was performed using BATS software (version 4.07)^55^. The first few scans were routinely discarded to account for possible surface contamination of the target due to contact with ambient air between the graphitization and the AMS measurement. Measurement parameters such as ^12^C current and ^13^CH current were checked. Time and isobar corrections were made prior to validation. Normalization, correction for fractionation and background corrections were applied for each individual run by measuring the oxalic acid II NIST standard and the phthalic anhydride blanks.

## Electronic supplementary material

Supplementary Table S1
