# Supplementary material for: Radiocarbon dating minute amounts of bone (3–60 mg) with ECHoMICADAS
Source: Sci Rep. 2017 Aug 2;7:7141. doi: 10.1038/s41598-017-07645-3 (PMC5541129; doi:10.1038/s41598-017-07645-3)
Supplement: Supplementary file 1 — Supplementary Table S1 [file 41598_2017_7645_MOESM1_ESM.pdf]

Supplementary information

Radiocarbon dating minute amounts of bone (3 - 60 mg) with ECHoMICADAS

S. CERSOY[1,*], A. ZAZZO[1], J. ROFES[1], A. TRESSET[1], S. ZIRAH[3], C. GAUTHIER[2], E. KALTNECKER[2], F. THIL[2], N. TISNERAT-LABORDE[2]

Table S1: Radiocarbon dating of the VIRI samples prepared according to different protocols. Samples characteristics and size, graphitization results and radiocarbon dates are reported for each sample. * Protocols are designated with letters in agreement with [35]. Yield is estimated as the ratio (in percent) of the total amount of collagen recovered over the amount of initial bone used for extraction. Normalized yield are calculated for each sample as the ratio of the extraction yield on the average yield obtained with the F protocol. Graphitization parameters are obtained from the AGE 3 system. The carbon mass correspond to the amount of carbon kept for the target. As the AGE 3 has a cutoff of 1 mg all carbon masses are necessarily below 1 mg. AMS parameters are from ECHoMICADAS system. ECHo n° corresponds to the target numbers. ** These samples were transferred in the liquid state.

VIRI E																
PRETREATMENT						*GRAPHITIZATION*					*AMS*					
**Sample prep code**	**ECHo n°**	**Sample size (mg)**	**Protocol***	**Amount collagen (µg)**	**Yield (%)**	**Normalized yield**	**Extracted carbon mass (µg)**	**%C**	**%N**	**C/N**	**Carbon mass (µgC)**	**F ^{14}C**	**Error (1σ)**	**^{14}C age (BP)**	**error**	**$\delta^{13}C$ (‰)**
16003	1129.1.2	166.9	F	2539	19.8	1.0	1144	41.9	15.3	3.2	999	0.0070	0.0002	39900	230	-22.6
16002	1129.1.1	166.9	F	2515	19.8	1.0	1139	42.1	15.4	3.2	995	0.0072	0.0002	39600	240	-25.0
16009	1129.1.3	166.9	F	2544	19.8	1.0	1138	41.5	15.2	3.2	993	0.0071	0.0002	39800	240	-17.1
16010	1129.1.4	166.9	F	2440	19.8	1.0	1089	41.8	15.2	3.2	982	0.0089	0.0002	37900	190	-20.4
16058	1129.1.5	166.9	F	2054	19.8	1.0	932	34.0	12.4	3.2	932	0.0077	0.0003	38100	810	-14.5
16059	1129.1.6	166.9	F	1919	19.8	1.0	869	42.2	15.4	3.2	869	0.0085	0.0004	37500	760	-13.2
16060	1129.1.7	166.9	F	1697	19.8	1.0	759	42.2	15.4	3.2	758	0.0087	0.0003	37100	720	-11.4
16061	1129.1.8	166.9	F	1361	19.8	1.0	599	42.3	15.4	3.2	600	0.0094	0.0002	36500	670	-13.9
16062	1129.1.9	166.9	F	1150	19.8	1.0	531	43.3	15.7	3.2	531	0.0091	0.0002	37800	3040	-14.1
16075	1129.1.10	166.9	F	1160	19.8	1.0	525	42.6	15.4	3.2	525	0.0097	0.0003	36400	670	-18.6
16237	1130.1.28	155.5	E	2521	17.6	0.9	1238	44.5	16.2	3.2	998	0.0070	0.0005	39800	600	-23.0
16232	1129.1.25	144.2	E	2362	14.0	0.7	1121	43.8	15.9	3.2	992	0.0092	0.0005	37600	440	-22.1
16238	1130.1.29	155.5	E	2536	17.6	0.9	1224	44.9	16.4	3.2	989	0.0067	0.0005	40300	570	-17.7
16239	1130.1.30	155.5	E	2345	17.6	0.9	1135	44.9	16.4	3.2	989	0.0074	0.0005	39400	540	-20.3
16234	1129.1.27	144.2	E	2310	14.0	0.7	1096	43.8	15.9	3.2	988	0.0083	0.0005	38500	480	-16.0
16233	1129.1.26	144.2	E	2370	14.0	0.7	1112	43.8	15.9	3.2	985	0.0078	0.0005	39000	510	-19.9
16194	1129.1.18	143.4	C	2276	10.5	0.5	1110	44.9	16.1	3.2	1000	0.0064	0.0004	40500	560	-27.6
16205	1129.1.21	143.4	C	2723	10.5	0.5	1314	44.8	16.2	3.2	998	0.0065	0.0005	40500	560	-22.5
16204	1129.1.20	143.4	C	2230	10.5	0.5	1086	44.9	16.2	3.2	996	0.0060	0.0004	41100	590	-18.5
16206	1129.1.22	143.4	C	2525	10.5	0.5	1215	44.7	16.2	3.2	990	0.0062	0.0004	40800	570	-14.4
16219	1129.1.24	143.4	C	2395	10.5	0.5	1157	44.9	16.3	3.2	990	0.0065	0.0005	40500	620	-21.5
16218	1129.1.23	143.4	C	2482	10.5	0.5	1216	44.8	16.3	3.2	989	0.0062	0.0005	40800	640	-19.7
16195	1129.1.19	143.4	C	2413	10.5	0.5	1168	44.8	16.2	3.2	984	0.0065	0.0005	40400	560	-25.0
16193	1129.1.17	143.4	C	2400	10.5	0.5	1168	44.8	16.2	3.2	984	0.0070	0.0005	39900	530	-23.8
16252**	1129.1.32	25.5	B	1662	21.3	1.1	720	40.7	14.7	3.2	720	0.0089	0.0006	37900	560	-11.5
16251**	1129.1.31	25.5	B	1380	21.3	1.1	593	40.6	14.6	3.2	593	0.0078	0.0006	39000	630	-20.3

16250**	1129.1.30	25.5	B	1072	21.3	1.1	434	39.0	13.9	3.3	434	0.0095	0.0006	37400	520	-22.3
16249**	1129.1.29	25.5	B	799	21.3	1.1	338	41.1	14.5	3.3	338	0.0104	0.0006	36700	490	-18.5
16248**	1129.1.28	25.5	B	529	21.3	1.1	216	40.4	14.1	3.3	216	0.0126	0.0007	35100	430	-22.5
17001**	1129.1.33	13	B	2777	21.4	1.1	1233	41.3	15.0	3.2	987	0.0058	0.0009	41400	1230	-17.8
17002**	1129.1.34	10	B	1940	19.4	1.0	849	41.3	15.0	3.2	849	0.0063	0.0009	40700	1150	-18.3
17003**	1129.1.35	7.2	B	1136	15.8	0.8	511	42.8	15.4	3.2	511	0.0072	0.0009	39700	1020	-18.7
17004**	1129.1.36	6.6	B	1301	19.7	1.0	575	41.9	15.1	3.2	575	0.0073	0.0009	39500	1010	-25.6
17005**	1129.1.37	5.4	B	691	12.8	0.6	298	41.7	14.9	3.3	299	0.0069	0.0015	40000	1700	-30.1
														**Mean**	**St dev**	
													**This work**	39032	1594	
													**Consensus**[44]	38772	2532	

VIRI H

PRETREATMENT						***GRAPHITIZATION***					***AMS***					
**Sample prep code**	**ECHo n°**	**Sample size (mg)**	**Protocol***	**Amount collagen (µg)**	**Yield (%)**	**Normalized yield**	**Extracted carbon mass (µg)**	**%C**	**%N**	**C/N**	**Carbon mass (µgC)**	**F ^{14}C**	**Error (1σ)**	**^{14}C age (BP)**	**error**	**δ^{13}C (‰)**
16011	1130.1.3	153.8	F	2689	12.6	0.8	1147	40.1	14.5	3.2	984	0.3059	0.0011	9510	30	-16.3
16012	1130.1.4	153.8	F	2590	12.6	0.8	1105	40.1	14.4	3.2	998	0.3021	0.0011	9610	30	-14.1
16005	1130.1.2	153.8	F	2481	12.6	0.8	1074	40.4	14.6	3.2	986	0.3089	0.0010	9440	30	-12.4
16004	1130.1.1	153.8	F	2466	12.6	0.8	1085	41.6	15.1	3.2	997	0.3050	0.0011	9540	30	-13.7
16063	1130.1.5	153.8	F	2175	12.6	0.8	964	41.3	14.9	3.2	964	0.3050	0.0014	9 540	40	-8.6
16064	1130.1.6	153.8	F	1803	12.6	0.8	797	41.3	14.9	3.2	797	0.3032	0.0014	9 590	40	-5.5
16065	1130.1.7	153.8	F	1570	12.6	0.8	688	41.4	14.9	3.3	688	0.3023	0.0014	9 610	40	-8.3
16067	1130.1.9	153.8	F	1163	12.6	0.8	519	41.8	15.0	3.3	519	0.3031	0.0015	9 590	40	-15.6
16066	1130.1.8	153.8	F	1362	12.6	0.8	615	42.0	15.1	3.2	616	0.3042	0.0014	9 560	40	-8.6
16071	1130.1.12	153.8	F	1153	12.6	0.8	515	41.6	14.8	3.3	515	0.3042	0.0014	9 560	40	-8.4
16196	1130.1.14	153.2	F	2250	22.8	1.4	998	41.2	14.8	3.2	998	0.3002	0.0012	9670	30	-14.5
16199	1130.1.17	153.2	F	2350	22.8	1.4	1034	40.9	14.7	3.2	998	0.3027	0.0012	9600	30	-12.1
16198	1130.1.16	153.2	F	2395	22.8	1.4	1052	41.2	14.8	3.2	997	0.3027	0.0012	9600	30	-17.1

16197	1130.1.15	153.2	F	2235	22.8	1.4	992	41.1	14.8	3.2	992	0.3035	0.0012	9580	30	-15.4
16207	1130.1.18	153.2	F	2413	22.8	1.4	1079	41.2	14.9	3.2	990	0.3041	0.0013	9560	30	-17.2
16227	1130.1.27	124.3	E	2318	12.7	0.8	1084	43.6	15.7	3.2	995	0.3027	0.0011	9600	30	-11.0
16225	1130.1.25	124.3	E	2266	12.7	0.8	1066	43.7	15.8	3.2	993	0.3020	0.0011	9620	30	-12.1
16226	1130.1.26	124.3	E	2230	12.7	0.8	1044	43.7	15.8	3.2	988	0.3030	0.0012	9590	30	-15.0
16208	1130.1.19	169.9	C	2335	18.9	1.2	1109	43.9	15.9	3.2	999	0.3065	0.0012	9500	30	-15.7
16209	1130.1.20	169.9	C	2264	18.9	1.2	1083	44.0	16.0	3.2	993	0.3059	0.0013	9510	30	-18.2
16224	1130.1.24	169.9	C	2344	18.9	1.2	1122	44.1	16.0	3.2	992	0.3037	0.0012	9570	30	-18.6
16210	1130.1.21	169.9	C	2497	18.9	1.2	1192	44.0	16.0	3.2	989	0.3040	0.0012	9560	30	-13.4
16223	1130.1.23	169.9	C	2331	18.9	1.2	1117	44.0	16.0	3.2	988	0.3036	0.0012	9570	30	-19.2
16211	1130.1.22	169.9	C	2515	18.9	1.2	1194	44.1	16.1	3.2	987	0.3040	0.0012	9570	30	-12.0
17006B**	1130.1.37	58.7	B	2517	15.7	1.0	1090	40.5	14.6	3.2	999	0.3032	0.0016	9 590	40	-10.1
17006A**	1130.1.36	58.7	B	2904	15.7	1.0	1041	33.8	12.2	3.2	987	0.3023	0.0016	9 610	40	-7.7
17006C**	1130.1.38	58.7	B	1782	15.7	1.0	773	40.8	14.6	3.3	773	0.3041	0.0018	9 560	50	-18.5
17006D**	1130.1.39	58.7	B	1055	15.7	1.0	467	42.9	15.2	3.3	467	0.3054	0.0015	9 530	40	-20.2
17006E**	1130.1.40	58.7	B	703	15.7	1.0	291	40.8	14.3	3.3	291	0.3044	0.0018	9 560	50	-15.0
17007**	1130.1.31	26.9	B	3635	13.1	0.8	1478	38.2	13.7	3.2	999	0.3036	0.0016	9 580	40	-11.2
17008**	1130.1.32	22.5	B	3998	17.1	1.1	1545	36.4	13.2	3.2	1000	0.3031	0.0017	9 590	40	-11.7
17009**	1130.1.33	15.5	B	2323	14.1	0.9	971	40.7	14.6	3.3	972	0.3019	0.0015	9 620	40	-4.6
17010**	1130.1.34	11.1	B	1881	16.1	1.1	721	37.9	13.5	3.3	721	0.3065	0.0017	9 500	40	-12.4
17011**	1130.1.35	8.9	B	15.3	25.4	1.6	282	39.7	14.0	3.3	283	0.3071	0.0019	9 480	50	-16.5
														**Mean**	**St dev**	
													**This work**	9567	46	
													**Consensus[44]**	9510	158	

VIRI I

PRETREATMENT						*GRAPHITIZATION*					*AMS*					
**Sample prep code**	**ECHo n°**	**Sample size (mg)**	**Protocol***	**Amount collagen (µg)**	**Yield (%)**	**Normalized yield**	**Extracted carbon mass (µg)**	**%C**	**%N**	**C/N**	**Carbon mass (µgC)**	**F ^{14}C**	**Error (1σ)**	**^{14}C age (BP)**	**error**	**δ^{13}C (‰)**
17012A	1231.1.11	167.3	F	1950	22.8	1.0	874	42.1	15.2	3.2	874	0.3524	0.0015	8380	30	-17.0
17012B	1231.1.12	167.3	F	1633	22.8	1.0	733	42.4	15.2	3.3	733	0.3523	0.0016	8380	40	-22.0
17012C	1231.1.13	167.3	F	1126	22.8	1.0	495	42.1	15.1	3.3	495	0.3525	0.0015	8380	40	-19.4
17012D	1231.1.14	167.3	F	831	22.8	1.0	361	42.8	15.0	3.3	361	0.3547	0.0016	8330	40	-13.1
17012E	1231.1.15	167.3	F	537	22.8	1.0	235	43.5	15.1	3.4	235	0.3440	0.0022	8570	50	-15.3
16253**	1231.1.1	22.8	B	526	22.4	1.0	214	41.2	14.6	3.3	214	0.3544	0.0019	8340	40	-28.4
16254**	1231.1.2	22.8	B	828	22.4	1.0	328	38.9	13.9	3.3	328	0.3510	0.0016	8410	40	-19.3
16255**	1231.1.3	22.8	B	1032	22.4	1.0	435	40.2	14.5	3.2	435	0.3495	0.0015	8450	40	-15.0
16256**	1231.1.4	22.8	B	1418	22.4	1.0	547	37.4	13.7	3.2	547	0.3492	0.0015	8450	40	-15.0
16257**	1231.1.5	22.8	B	1465	22.4	1.0	598	38.8	14.1	3.2	598	0.3546	0.0018	8330	40	-13.4
17013**	1231.1.9	14.2	B	3540	24.1	1.1	1545	40.9	15.0	3.2	991	0.3502	0.0017	8430	40	-6.8
17014**	1231.1.10	9.4	B	1995	21.1	0.9	814	39.2	14.3	3.2	815	0.3529	0.0017	8370	40	-12.9
16280**	1231.1.6	6.4	B	1843	28.1	1.3	670	33.9	12.2	3.2	670	0.3550	0.0000	8320	40	-19.6
16293**	1231.1.7	4.2	B	1030	24.1	1.1	285	26.6	9.5	3.2	285	0.3538	0.0000	8350	50	-19.3
16305**	1231.1.8	2.8	B	892	31.1	1.4	237	26.3	9.3	3.3	237	0.3522	0.0000	8380	50	-21.0
														**Mean**	**St dev**	
													**This work**	8391	65	
													**Consensus**[44]	8328	176	

VIRI F

PRETREATMENT						***GRAPHITIZATION***					***AMS***					
**Sample prep code**	**ECHo n°**	**Sample size (mg)**	**Protocol***	**Amount collagen (µg)**	**Yield (%)**	**Normalized yield**	**Extracted carbon mass (µg)**	**%C**	**%N**	**C/N**	**Carbon mass (µgC)**	**F ^{14}C**	**Error (1σ)**	**^{14}C age (BP)**	**error**	**δ^{13}C (‰)**
16212	1211.1.1	590	F	2472	23.9	1.0	1094	41.7	15.3	3.19	986	0.7257	0.0021	2580	20	-16.5
16236	1211.1.7	590	F	2430	23.9	1.0	1107	42.0	15.4	3.19	997	0.7315	0.0021	2510	20	-21.9
16241	1211.1.9	590	F	2380	23.9	1.0	1079	42.1	15.4	3.19	989	0.7299	0.0020	2530	20	-19.5
16220	1211.1.3	590	F	2364	23.9	1.0	1044	40.9	15.0	3.19	990	0.7286	0.0020	2540	20	-15.3
16235	1211.1.6	590	F	2328	23.9	1.0	1036	41.4	15.2	3.18	982	0.7284	0.0020	2550	20	-17.9
16221	1211.1.4	590	F	2294	23.9	1.0	1037	41.9	15.3	3.20	983	0.7317	0.0020	2510	20	-18.7
16240	1211.1.8	590	F	2275	23.9	1.0	1022	41.9	15.4	3.18	987	0.7318	0.0021	2510	20	-21.9
16213	1211.1.2	590	F	2260	23.9	1.0	1010	41.4	15.1	3.20	993	0.7285	0.0021	2540	20	-16.5
16222	1211.1.5	590	F	2168	23.9	1.0	986	42.0	15.3	3.19	987	0.7284	0.0020	2550	20	-21.0
14384A	1211.1.23	590	F	2215	23.9	1.0	962	42.4	15.5	3.20	961	0.7302	0.0027	2530	30	-15.2
14384B	1211.1.24	590	F	1904	23.9	1.0	840	42.3	15.4	3.20	841	0.7315	0.0033	2510	40	-18.2
14384Pré	1211.1.22	590	F	1872	23.9	1.0	811	42.5	15.5	3.20	811	0.7294	0.0028	2540	30	-14.3
14384C	1211.1.25	590	F	1309	23.9	1.0	579	42.0	15.2	3.20	580	0.7279	0.0027	2550	30	-23.0
14384D	1211.1.26	590	F	857	23.9	1.0	368	42.8	15.3	3.30	368	0.7324	0.0024	2500	30	-21.9
16266**	1211.1.14	26	B	1560	19.6	0.8	669	40.6	14.7	3.22	669	0.7328	0.0023	2500	30	-17.5
16265**	1211.1.13	26	B	1404	19.6	0.8	594	39.9	14.3	3.25	594	0.7282	0.0023	2550	30	-18.0
16264**	1211.1.12	26	B	1186	19.6	0.8	504	40.4	14.5	3.25	504	0.7302	0.0023	2530	30	-17.1
16263**	1211.1.11	26	B	888	19.6	0.8	373	40.8	14.6	3.27	373	0.7312	0.0024	2520	30	-20.4
16262**	1211.1.10	26	B	531	19.6	0.8	218	41.1	14.6	3.29	218	0.7326	0.0028	2500	30	-29.0
17016**	1211.1.20	12.3	B	2439	19.8	0.8	1092	42.1	15.3	3.20	986	0.7244	0.0029	2590	30	-15.2
17015**	1211.1.21	10.3	B	1441	14.0	0.6	626	40.3	15.0	3.13	626	0.7284	0.0025	2550	30	-16.1
16270**	1211.1.15	26	B	2495	19.6	0.8	1127	41.6	15.3	3.18	997	0.7303	0.0023	2530	30	-18.9
16296**	1211.1.16	6.8	B	2107	13.2	0.6	713	31.5	11.3	3.24	713	0.7278	0.0000	2550	30	-18.6
16311**	1211.1.17	4.5	B	1059	23.5	1.0	303	24.6	10.0	2.86	304	0.7324	0.0000	2500	30	-16.9
														**Mean**	**St dev**	
													**This work**	2532	25	
													**Consensus**[44]	2525	69	
